# The Role of Chair Design in Dental Ergonomics: A Kinematic Assessment of Movement and Ergonomic Risk

**DOI:** 10.3390/bioengineering12040353

**Published:** 2025-03-29

**Authors:** Fabian Holzgreve, Jasmin Preuß, Christina Erbe, Werner Betz, Eileen M. Wanke, Gerhard Oremek, Doerthe Brueggmann, Albert Nienhaus, David A. Groneberg, Christian Maurer-Grubinger, Daniela Ohlendorf

**Affiliations:** 1 Institute of Occupational Medicine, Social Medicine and Environmental Medicine, Goethe University Frankfurt, 60590 Frankfurt am Main, Germany; 2 Department of Orthodontics, University Medical Centre of the Johannes Gutenberg University, 55131 Mainz, Germany; 3 Institute of Dentistry, Goethe University Frankfurt, 60590 Frankfurt am Main, Germany; 4 Principles of Prevention and Rehabilitation Department (GPR), Institute for Statutory Accident Insurance and Prevention in the Health and Welfare Services (BGW), 22089 Hamburg, Germany

**Keywords:** RULA, musculoskeletal disorders, inertial motion capture, inertial motion units, statistical parametric mapping

## Abstract

Introduction: Dental professionals are susceptible to musculoskeletal disorders due to unphysiological postures during treatment, which can be mitigated by the choice of a work chair to improve ergonomic working posture. Methods: In this study, the influence of five different work chairs on the ergonomic risk assessment using RULA and the working behaviour of 22 right-handed dentists was investigated. To this end, dental treatment was simulated on a phantom head, with the body posture recorded using an inertial motion capture system. The resulting kinematic data were converted into a continuous RULA scheme, and statistical methods (Friedman test with Conover–Iman comparisons and statistical parametric mapping) were used to compare the chairs. The significance level was set at *p* ≤ 0.05. Results: The RULA analysis revealed no significant differences between the task chairs that were tested; however, it should be noted that all of the task chairs exhibited an increased ergonomic risk (RULA ≥ 5), which indicates an increased risk of musculoskeletal disorders. Significant multiple comparisons (SPM analysis) between the selected chairs of the relative occurrence of total joint angles were between Chair 1 vs. 5 (*p* ≤ 0.03 for shoulder left flexion–extension), Chair 2 vs. 4 (*p* ≤ 0.03 for shoulder right flexion–extension), Chair 1 vs. 3 (*p* ≤ 0.03 for trunk right flexion–extension, 0.04 for trunk lateral flexion and 0.05 for elbow left flexion–extension), Chair 3 vs. 4 (*p* ≤ 0.05 for shoulder left flexion–extension and 0.01 shoulder right flexion–extension), and between Chair 2 vs. 3 (*p* ≤ 0.05 for elbow left flexion–extension). Discussion: The study’s findings indicate that the selection of work chairs did not have a significant impact on the ergonomic risk, which remained consistently high across all the chairs. Nevertheless, the analysis of joint angles demonstrated that the Ghopec chair was more frequently associated with greater joint angles, with only a few significant deviations. However, it should be noted that these significant differences in joint angles occurred only sporadically, did not demonstrate a clear and consistent trend across all the chairs, and have no clinical impact. Overall, the results confirm that the working posture of the dentists has a potential risk of developing musculoskeletal disorders, while the ergonomic design of the work chairs plays a rather subordinate role.

## 1. Introduction

The prevalence of musculoskeletal disorders (MSDs) in dentistry and the resulting days of incapacity for work are high [1]. In terms of musculoskeletal disorders, 68–100% of dental professionals are affected, particularly the lower back (29–94.6%), shoulders (25–92.7%), and neck (26–92%) [2]. This is due to unfavourable and static postures as the main causes of MSDs in dentistry, as both dentists and their assistants often adopt an unfavourable posture in order to have a clear view of the patient’s mouth while ensuring access to dental instruments and trays [3,4,5,6]. These findings emphasise the need for workplace-related measures, which are of particular importance in the context of implementing and optimising preventive measures. Lietz et al. [7] analysed effective prevention strategies of MSDs in their systematic literature review and ended up analysing five studies with magnifying loupes or prism glasses, three on ergonomic training, two studies on dental chairs, and one study on dental instruments. Although the ergonomic measures had a positive effect on the study results in all the studies, the small number of studies must be taken into account. In another systematic review [8], four studies were also identified with regard to this problem, with the quintessence that none could prove a successful reduction in MSDs through a saddle chair. In addition to the saddle chair, there are also other ergonomic dental chair concepts as a preventive measure against MSDs. However, to date, there have only been a few studies that systematically compare the influence of different chair concepts on working posture and ergonomic risk. When analysing the current research situation, the heterogeneity of the studies adds to the difficulty due to the heterogeneous selection of methods and the variability of the stool concepts investigated. For instance, studies have used strain gauges [9,10], electromyography (EMG) [9,10,11], video stereography [12], or RULA-based photographic analyses [13,14], each of which captures distinct aspects of posture and muscle activity. The studies also vary in terms of chair concepts analysed; while some studies only compared two chair concepts (e.g., Dable et al. [13] and Gandavadi et al. [14]: saddle chair vs. standard chair), others analysed up to six different models with varying seat surfaces and backrests [11,12]. The resulting variety of chair concepts further contributes to the inconsistency of the findings [8]. For instance, de Bruyne et al. [10] found a positive effect of the Ghopec chair on working posture, while Fiedler [11] and Huppert et al. [12] found no significant influence of chair choice on posture or muscle activity. Conversely, Dable et al. [13] and Gandavadi et al. [14] and the systematic review by Plessas et al. [8] demonstrated the merits of ergonomic saddle chairs with respect to working posture. Based on the current state of research, the question of the extent to which the ergonomic layout of a dental chair influences working posture and ergonomic risk during dental procedures remains unresolved. The results to date are inconsistent and are mainly based on short measurement sequences, which emphasises the need for further research. There is a lack of analyses on the influence of chair layout that consider the entire body posture over an entire dental treatment, especially in view of the high prevalence of MSDs of the entire body [2]. In addition, the question of the extent to which the ergonomic layout of a work chair influences working posture and ergonomic risk during dental procedures remains unresolved. Studies that have already been carried out have also not been analysed rom an ergonomic, occupational health perspective. The ergonomic assessment of working conditions is a key aspect in the prevention of work-related musculoskeletal disorders, particularly within occupational groups characterised by elevated physical stress, such as dentistry. A proven and frequently employed tool for ergonomic risk assessment is the Rapid Upper Limb Assessment (RULA) [15], a method particularly utilised within the healthcare sector, enabling expeditious evaluation of potential ergonomic risks [16]. While RULA was originally designed as a paper–pencil method and requires a trained person for assessment [17], modern technologies now enable objective recording of movement sequences through the use of inertial measurement systems [18]. This innovative approach was first described in the SOPEZ (Study for the Optimisation of Ergonomics in the Dental Practice) project [6] and has already been used in several studies [19,20,21,22,23]. RULA analysis using inertial measurement systems offers significant advantages over the classical method. While the conventional method only captures snapshots, the digital variant allows continuous measurement over the entire working period [18,24]. This methodology provides a comprehensive and realistic data acquisition, whilst eliminating subjective judgements by observers, as the system generates objective kinematic data [24,25]. Furthermore, the whole-body measurement enables a detailed analysis of ergonomic risks, which could only be recorded to a limited extent with selective observation [24,25,26]. A further methodological advantage of this study is the use of a standardised measurement protocol on the phantom head, which ensures a high degree of comparability of the measured values [19,20,21,22,23]. The long measurement duration of over 30 min also allows long-term stress patterns to be recorded, which are difficult to identify using the classic RULA method [19,20,21,22,23].

The objective of this study is, therefore, to systematically analyse the influence of five different work chair designs on the ergonomic risk and relative joint angle occurrence during dental treatment. For more precise analysis using RULA, the analysis considers the two halves of the body separately due to the asymmetrical nature of the work. Therefore, the following hypotheses are tested:

**Hypothesis** **1:** *A significantly different ergonomic risk can be determined between the chairs based on RULA.*

**Hypothesis** **2:** *A significantly different posture can be determined between the chairs based on relative joint angle occurrence.*

## 2. Material and Methods

### 2.1. Subjects

In this study, 22 dentists (14 female and 8 male) aged between 22 and 65 years (mean age: 32.86 years; standard deviation: 8.67 years) with a professional experience of 1–38 years (median of 5 years and interquartile range of 2 years) participated voluntarily. The median height of the study participants was 174.36 cm ± 9.94 cm, and their weight was 68.72 kg ± 12.60 kg. The mean body mass index (BMI) of the female participants was 21.39 ± 2.11, while the BMI of the male participants was 24.34 ± 2.76. The inclusion criteria comprised a German licence to practise medicine, age between 18 and 65 years and right-handedness.

The exclusion criteria for participation included left-handedness, recent musculoskeletal injuries or surgical interventions within the last six months, rheumatic diseases, severe deformities of the spine (e.g., scoliosis) and stiffened spinal joints (pathological or surgical), genetic neurological diseases or muscle diseases, and the daily use of painkillers and muscle relaxants. The exclusion criteria were handed out and asked about in a separate sheet. The participants who did not fulfil the inclusion criteria were excluded from participation in this study.

Informed and written consent was obtained from all the subjects.

The study was conducted in accordance with the Declaration of Helsinki, and the protocol was approved by the Ethics Committee of the Goethe University Frankfurt am Main (356/17) on [22 December 2021].

### 2.2. Dental Working Chairs

**Figure 1:**
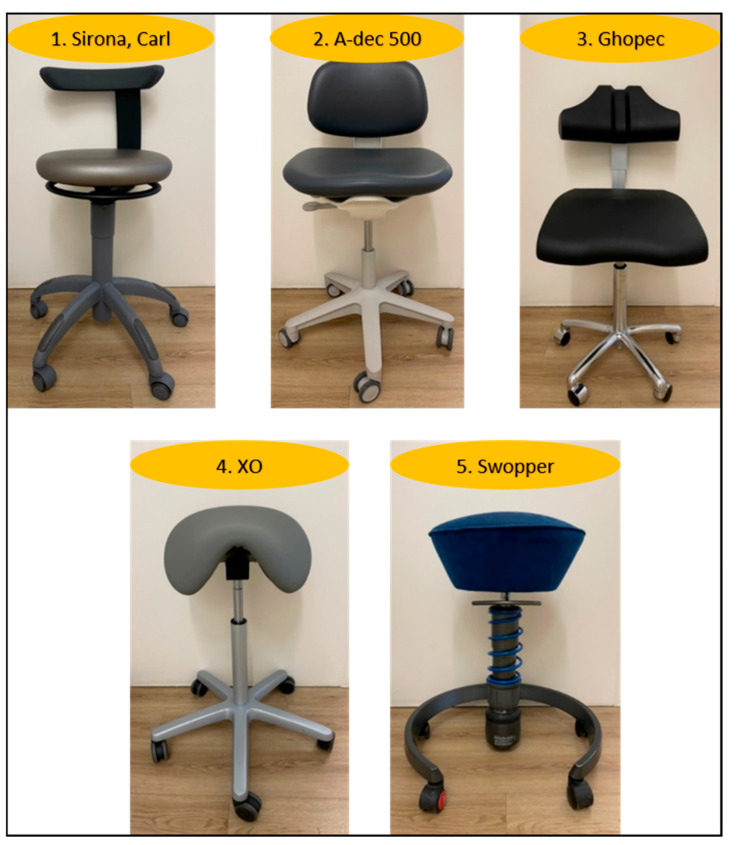
Types of chairs used: 5 different ergonomic chairs used by professional dentists were measured. Chair 1: Dentsply Sirona (Germany), CARL; Chair 2: Newberg Oregon, USA, A-dec; Chair 3: Veenendaal, Netherlands, Ghopec BV; Chair 4: Hørsholm, Denmark, XO- Saddle chair; Chair 5: Haar, Germany, Aeris, Swopper.

In this study, five distinct chairs were selected for analysis. These chairs varied in their design and underlying ergonomics. The characteristics of these chairs are outlined in Table 1, and each seating concept is further illustrated in Figure 1.

The selection of Chairs 1, 2, 3 and 4 was made on the basis that they are the models most commonly utilised in German dental practices and universities. Conversely, Chair 5, the Aeris Swopper, is not designed for the field of dentistry. It is an ergonomically designed chair whose construction is sufficiently flexible to allow for ‘moving sitting’ (bouncing), thereby enabling the muscles of the body to be moved unconsciously, thus actively reducing a static sitting posture.

### 2.3. Inertial Motion Capture

The collection of kinematic data was facilitated by the utilisation of the MVN Link inertial measurement system (Movella Technologies B.V., Enschede, Netherlands). Each participant was outfitted with a full-body suit designed to ensure a close fit, fabricated from Lycra, to which 17 sensors were attached in a manner that precluded slippage. The sampling rate was set at 240 Hz, and the measurement error was specified by the manufacturer as ±1%.

The sensors are small inertial measuring units with integrated gyroscopes, accelerometers, magnetometers, and barometers, which can be used to measure angular velocities, accelerations and gravity, the magnetic field of the earth, and air pressure [27].

As the measurement was carried out in a seated position on dental chairs, the measurement was performed in the ’no-level’ setting. This ensured that the pelvis remained at a constant height, thereby serving as a fixed point for the movements in the individual coordinate system. The MATLAB R2024a programme, developed by MathWorks (Natick, MA, USA), was utilised for the processing of the raw data.

### 2.4. Study Protocol

**Figure 2:**
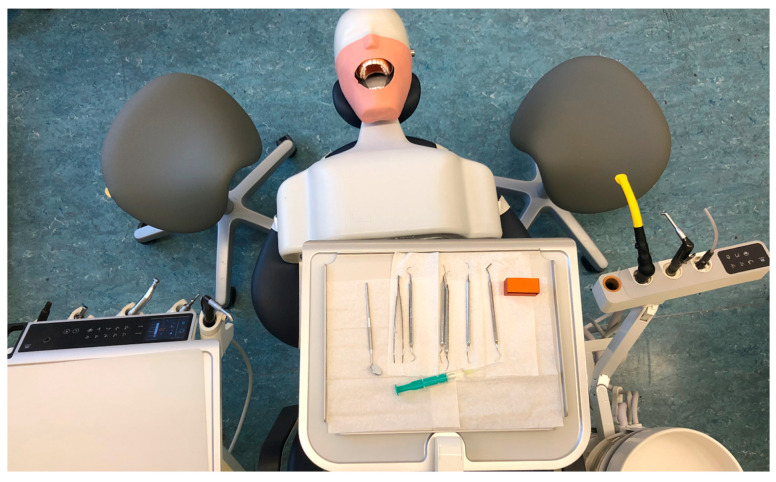
Basic concept 1 according to Kimmel. The dentist is seated at 9 o’clock, looking directly into the patient’s mouth and holding instruments to the right. The dental assistant is seated opposite at 3 o’clock, reaching for instruments on the left and for the cabinet on the right. The instruments are low and the tray is above the patient’s chest.

The biomechanical measurement was conducted on a dental treatment centre that was configured in accordance with Kimmel’s fundamental concept 1 (Figure 2) [28]. The rotating dental instruments, the multifunctional syringe, and the scaler were positioned to the right of the patient, with the assistant’s treatment instruments positioned to the left. The dentist’s typical seating position is between 9 and 10 o’clock, while the assistant’s is between 1 and 3 o’clock [29]. This configuration is observed in 88.1% of the dental practices in Germany [30].

According to the manufacturer’s instructions, each test subject was instructed in the use of the various dental chairs, after which the chairs were adjusted. For the chairs designated 1, 2, 3, and 5, no such instructions were available, necessitating the development of a methodology to effectively control the influence of seat height on posture. This was achieved by standardising the angle between the upper and lower leg to 120°, thereby enabling the adjustment of seat height to different body heights.

Following the completion of the individual adjustments to each chair, the MVN Link system was installed and subsequently calibrated to align with the anthropometric characteristics of each subject. Each dentist was tasked with performing the following four dental procedures on the dummy head, with each task being executed in a distinct quadrant (Table 2). The dummy head was affixed to the treatment centre, with plastic practice teeth (Morita, J. Morita Corp., Osaka, Japan) screwed into the upper and lower jaw models. A plastic mask was employed to simulate the patient’s mouth on the phantom head, and the models were covered with a gum mask.

The subjects were assisted by a dental assistant in the execution of the tasks. However, the activities of the assistant were not documented, but rather served to simulate the actual working conditions in a realistic manner. The classic sub-areas in dentistry are tooth preservation, orthodontics, surgical dentistry, and dental prosthetics. In their day-to-day work, general dentists deal with the sub-areas of conservative dentistry and prosthodontics. This includes all tasks that are used in our standardised protocol: tasks 1, 2, and 4 are part of conservative dentistry or tooth preservation, while task 3 belongs to the dental prosthodontics field. The selected tasks, therefore, reflect the everyday work of general dentists. Due to the fact that the aim of the present study focused on the posture of the dentist on an ergonomic chair and not on the quality of the treatment, it was carried out on a dummy head. In this way, the same treatment conditions were ensured for each participant during each dental activity and the possible influence of external patient-specific factors on posture was minimised.

The four tasks were repeated on all five treatment chairs. The order of tasks and the position of the treatment chairs were randomised. All the study participants wore magnifying glasses during the measurements.

### 2.5. Analysis of the Kinematic Data

The Rapid Upper Limb Assessment (RULA) is a method of conducting a preliminary analysis and ergonomic assessment. The distribution of joint angles was analysed in order to detect even minor differences between postures on the chairs, with the aim of facilitating a more sensitive comparison of posture on different chairs. RULA was developed by McAtamney and Corlett in 1993 [15]. It is utilised to evaluate the ergonomic risk potential of workplaces associated with work-related musculoskeletal disorders of the upper extremity. The result of the assessment is an estimate of whether further investigations and ergonomic changes to the workplace are necessary. This quick review of work processes can be carried out by hand directly next to the workstation [31]. The assessment involves the documentation and evaluation of the strain on different body regions caused by postures and movements, the exertion of force, and the activity of muscles (static or repetitive movements) during a work process.

Postures are categorised as acceptable if they achieve a RULA score of 1–2. No alterations to the workplace are required in such cases. However, should the RULA score be between 3 and 4, a more thorough examination of the workplace is necessary, as changes may be required. If the RULA score is between 5 and 6, subsequent measures must be initiated in the near future. In the event of a score of 7, immediate measures must be taken [15].

**Figure 3:**
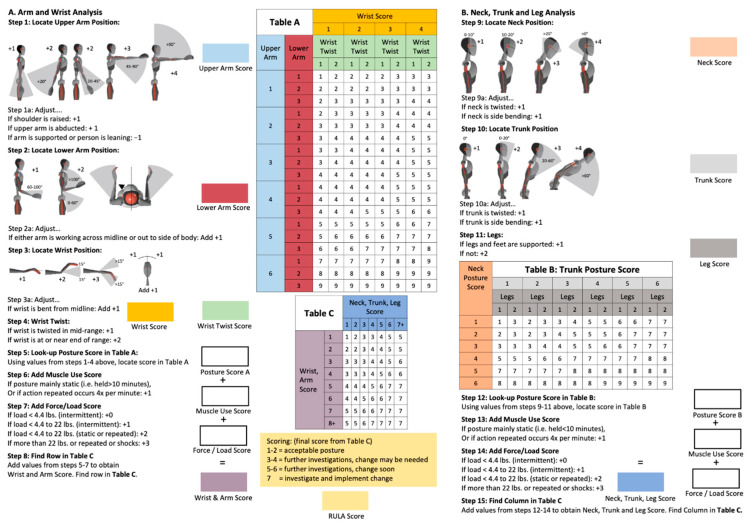
Assessment of posture and ergonomic stress using the RULA worksheet [18].

With direct measurement using an inertial measurement system, as in this study, the assessment is independent of subjective expert opinion due to the continuous recording of objective motion data. When observational methods are combined with direct measurement, the objective motion data can be used for ergonomic risk assessment. For this purpose, the script by Maurer-Grubinger et al. [18] can be used to automatically analyse motion data using RULA (Figure 3).

Specific steps or point values were selected for evaluation and the results of the different chairs were compared:

1. Final Score—RULA Step 15;
2. Neck Score—RULA Step 9;
3. Trunk Score—RULA Step 10;
4. Upper Arm Score (left and right)—RULA Step 1;
5. Lower Arm Score (left and right)—RULA Step 2;
6. Wrist Score (left and right)—RULA Step 3 + 4.

The following parameters were recorded for each chair:

- RULA median and interquartile range;
- Total RULA distribution of each RULA point;
- Relative joint angle occurrence.

### 2.6. Statistical Analysis

The study included 21 patients. Using a one-factorial analysis of variance with repeated measures, this number of cases results in: α = 5%; β = 80%; Cohen’s f = 0.25; number of groups (chairs) to be compared = 5; a correlation between the measurements of 0.54. The effect size and correlation are based on the data for the dentists in the study by Ohlendorf and colleagues [32]. The correlation between the measurements is expected to be similar, but the effect of the chair is estimated to be about twice as large as the effect of different treatment concepts on ergonomic risk. As a Friedman test is used to analyse the main outcome measure between the five associated groups, 10% is added to the calculated case number of n = 19, resulting in a case number of 21 patients. The number of cases was calculated using GPower 3.1.

In addition to Matlab, the BiAS programme (version 11.12, BiAS. for Windows, Epsilon Verlag, Darmstadt, Germany) was used for statistical analysis. The Kolmogoroff–Smirnoff–Lilliefors test was used to check for normal distribution, which showed that most of the parameters were not normally distributed, except for the height and weight of the participants. In order to test hypothesis 1, multiple related samples must be compared with each other and a post hoc test must be carried out. Due to the non-normal distribution, the Friedman test was used to compare chairs, followed by pairwise comparisons using the Conover–Iman test. The *p*-values were then subjected to a Bonferroni–Holm correction. In order to test hypothesis 2, inferential statistical methods for continuous data are necessary. For this purpose, Systematic Parametric Mapping (SPM) is used to analyse the distribution of joint angles between the different chairs [33,34,35]. The level of significance was *p* ≤ 0.05.

## 3. Results

The median of the final score was analysed separately for the right and left sides of the body (Table 3). For the right side of the body, the lowest value of five was measured on the XO saddle chair and the highest value of six on the A-dec 500 chair. The other chairs, Carl, Ghopec and Aeris, Swopper, were in between at 5.5 (Table 1). The median for the left side of the body was also in the second-highest range. It was 5 on the Carl, XO and Aeris, Swopper chairs, 5.5 on the A-dec 500 and 6 on the Ghopex.

Furthermore, the grouping by body region shows only marginal differences in the median. While there are no differences in the median for the neck, left lower arm, left wrist, right upper arm, and right lower arm regions, the trunk, left upper arm, and right wrist regions show small differences between the chairs around a median of 0.5.

Tests for differences showed no significant differences in the RULA scores between chairs in any region.

**Figure 4:**
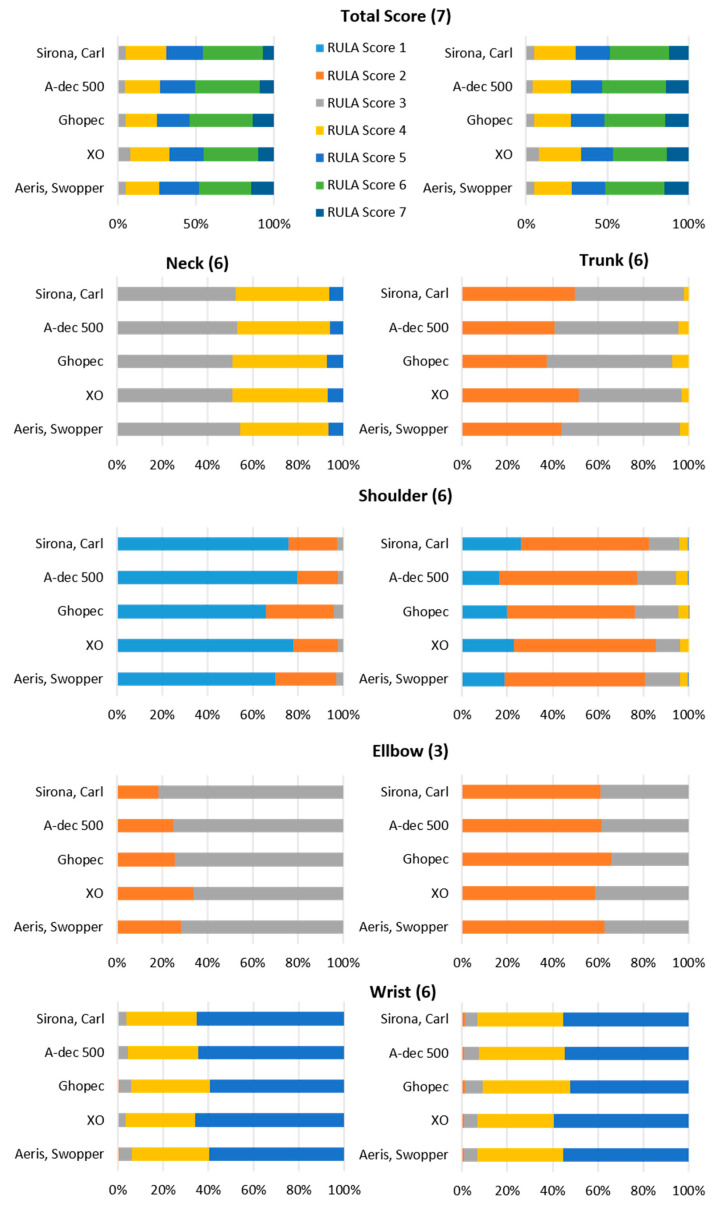
Relative proportions of the different RULA scores for all five chairs tested. The maximum possible RULA score of each region is specified in brackets.

Looking at the distribution of time spent in the different RULA scores, it is noticeable that on all chairs no time was spent in a posture categorised as acceptable, either for the left or the right side of the body (Figure 4). Less than 1/3 of the time was spent in postures considered to be in the low-risk range of final RULA scores three and four. Correspondingly, more than 2/3 of the time was spent in a posture classified as a health risk regardless of the type of stool. The highest overall score of final score seven, which recommends immediate action and change, was recorded on all the chairs in 7.21% to 14.76% of the time measured. On the ergonomic office chair Aeris, Swopper, the highest relative time in the highest RULA score was recorded with 14.72% for the right and 14.76% for the left side of the body. The lowest percentage of time in the highest RULA score of 11.76% for the right and 7.21% for the left side of the body was measured on the Carl chair, Sirona, with a horizontal seat.

The ergonomic loads on the neck differ only slightly between the dental chairs analysed. All the chairs have a very low proportion of high stress values (RULA 5), while the stresses are relatively evenly distributed between moderate values (RULA 3 and 4). Slight differences can be observed in the trunk score. Chairs 1 and 4 show a slightly higher proportion of low stress values (RULA 2) and correspondingly lower proportions of RULA 3. Chair 3, on the other hand, shows a slightly higher proportion of RULA 4, indicating a slightly higher stress in the trunk. A striking result can be seen when looking at the shoulders (upper arm score), where there is a strong lateral difference. The right shoulder is significantly more stressed than the left, although the overall stress in both shoulders can be classified as low, as RULA values 1 and 2 predominate. There are only minor differences between the chairs analysed.

The Ghopec and Aeris Swopper chair has a slightly higher load on the left side, while the Sirona, Carl, and XO models give slightly better results on the right side of the upper arm score. There are also lateral differences in the lower arm, but with the opposite load distribution compared to the shoulder. The ergonomic load on the left lower arm is higher, as the maximum score (RULA 3) is often reached. The total load on the upper arm is slightly higher than on the shoulder. Among the chairs analysed, the XO chair stands out positively on the left side, while the Sirona and Carl models have a slightly higher proportion of RULA 3 values. In contrast to the other joints of the upper extremity, there are only minimal lateral differences in the wrist score. However, the overall ergonomic risk is slightly higher here. All the chairs analysed show predominantly RULA values of four and five, indicating an increased level of stress. There are hardly any lower stress values in the wrists, neither on the left nor on the right side.

The analysis of the RULA values for the left upper arm score shows an overall low ergonomic risk, as the values are predominantly one. The exception is the Ghopec, which has slightly higher values with a median of 1.5, but no significant difference compared to the other chairs.

**Figure 5:**
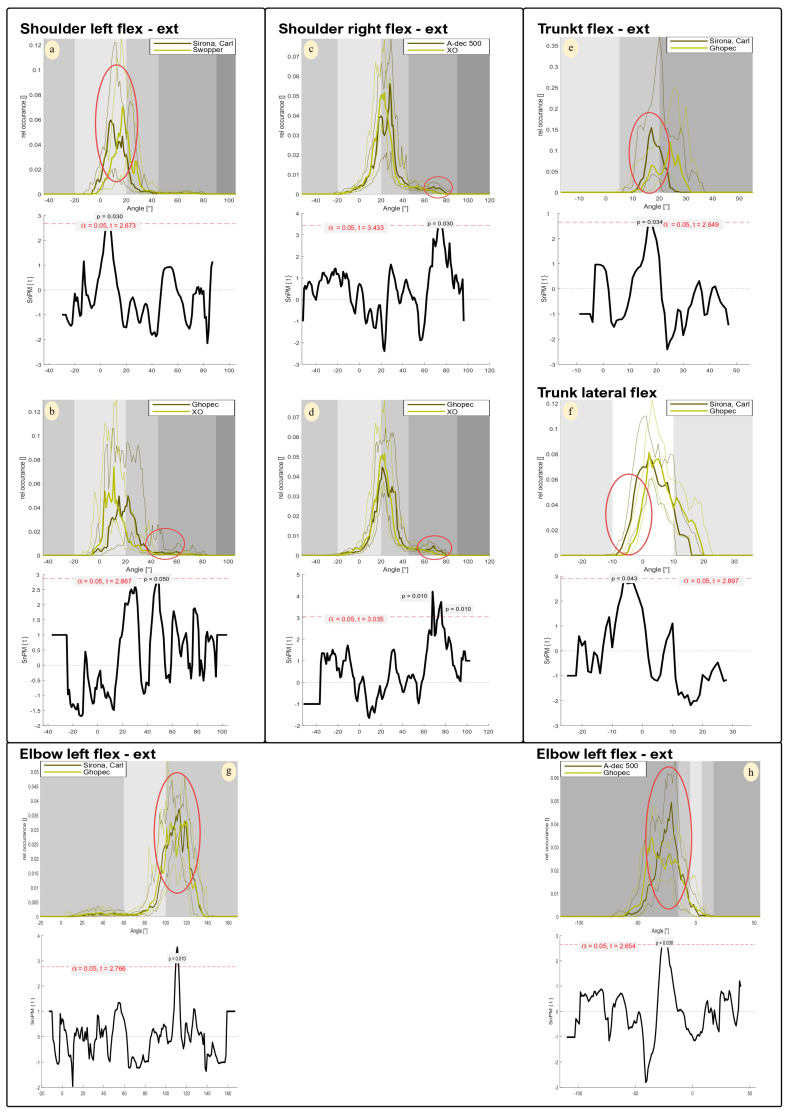
Significant multiple comparisons between selected chairs of the relative occurrence of total joint angles and corresponding SPM analysis. (**a**,**b**) left shoulder (sagittal plane); (**c**,**d**) right shoulder (sagittal plane); (**e**) trunk (sagittal plane); (**f**) trunk (frontal plane); (**g**,**h**): left elbow (sagittal plane). Top image: abscissa = joint angle; ordinate = relative occurrence; grayscale = RULA score; thick line = median values; thin lines = 1st/3rd quartile. Bottom image: abscissa = joint angle; ordinate = t-values; red line = significance threshold. The red circles mark significant areas of joint angle and relative occurrence between chairs.

A more differentiated analysis of the shoulder joint angles in the sagittal plane, which offers greater sensitivity and precision, reveals additional differences. This shows that the Swopper has a significantly higher proportion of larger flexion angles compared to the Sirona, Carl chair. However, these significant differences remain within the joint angle range of −20° to +20°, which corresponds to a RULA value of one (Figure 5a). Another significant difference can be seen when comparing Ghopec and XO. Ghopec has a higher proportion of flexion angles, with a significant proportion of these exceeding the RULA threshold of 20°. This is also reflected in a higher median of 1.5 for Ghopec (Figure 5b).

Significant differences in the distribution of joint angles were also observed for the right shoulder, particularly when comparing A-dec 500 and XO, and Ghopec and XO. It is noticeable that these differences do not occur in the most common angle ranges, but mainly in the extreme flexion range (Figure 5c,d). In particular, A-dec 500 shows significantly higher proportions of flexion angles around 70°, indicating increased stress in this angle range. Nevertheless, the differences observed remain within the relevant RULA limits, which are between 40° and 90° for this range (Figure 5d).

In trunk flexion, the relative distribution of joint angles shows significant differences between Sirona, Carl, and Ghopec, particularly in the areas with the highest probabilities of occurrence. Ghopec shows significantly higher proportions of larger flexion angles compared to Sirona, Carl (Figure 5e). This difference is not directly reflected in the RULA score (Table 1), but can be derived to a small extent from the RULA proportions (Figure 4).

The analysis of the lateral flexion of the trunk shows that Sirona, Carl has a higher proportion of lateral tilt to the left overall, while Ghopec has a higher proportion of lateral tilt to the right. Significant differences occur in the left inclination, with Sirona, Carl showing significantly higher proportions compared to Ghopec. At the same time, the largest angular proportions are in the area of lateral inclination to the right, with Ghopec tending to show higher values here. However, these differences are not significant (Figure 5f).

Differences can be seen in the joint angle proportions with greater elbow flexion on the left side. In particular, a significant difference can be seen in the range between 110° and 115°, with Sirona, Carl showing higher joint angle proportions (Figure 5g).

In wrist flexion, it is clear that Ghopec has a significantly higher proportion of negative joint angles (wrist extension). However, these differences are all within the range that results in a RULA score (Figure 5h).

## 4. Discussion

The aim of this study was to compare the ergonomic posture during dental treatment on different dental chairs for the first time by means of quantitative data collection and ergonomic evaluation. The results obtained demonstrate that there are no significant ergonomic differences in posture between the dental treatment chairs during dental activities (Table 3). Hypothesis no. 1 can be falsified. However, all the tasks on each of the five chairs exhibited an increased ergonomic risk (RULA ≥5), which indicates an increased risk of musculoskeletal disorders accompanied by the advice to carry out further investigations and implement changes immediately.

Due to the limited sensitivity of RULA, the joint angle frequency was also analysed in this study. This enabled a more detailed recording and detection of minor differences in posture. The advantages of this method over RULA in terms of sensitivity can be clearly seen in Figure 4. The graphs in different shades of grey illustrate at which joint angles RULA scores are assigned and which threshold values are relevant for the assignment of RULA scores. It is clear that the identified postural differences generally remain within a RULA score and would, therefore, have remained undetected by RULA. The majority of statistical significances observed in the analysis of relative joint angle occurrence indicate only marginal differences without clinical relevance or ergonomic influence (RULA score). In addition, this more detailed analysis reveals that significantly different curves were observed almost solely in the sagittal plane in the shoulder and trunk region. Hypothesis number 1 can, therefore, be verified, but it should be noted that the clinical relevance as mentioned above can be categorised as low. Chair 3, the Ghopec, was conspicuous here. The Ghopec chair is more frequently represented with an increased frequency in the area of larger joint angles in the few significant differences identified especially in the shoulder region (Figure 5b,d–f,h). The only joint angle that caused a different assessment of the ergonomic working method was registered when looking at the upper body, with flexion of 17° being adopted significantly more frequently (*p* = 0.034) on the Carl chair than on the Ghopec chair, where it was mostly 23°. Joint angles of 20° or less are rated with a lower score. Nevertheless, each of the four (apart from Chair 5 ‘Swopper’) dental treatment chairs (Chairs 1–4) is declared to be an ergonomic dental chair. However, the ergonomic concept of these chairs does not appear to (ergonomically) improve body posture during dental work. The examined dental chairs may be too similar in terms of design, cushioning, and height adjustment to detect relevant ergonomic differences. However, Chair 5 features a different layout, as it allows movement in all three planes and has a torsion spring mechanism that can be adjusted to the user’s body weight. Despite these differences, no significant ergonomic advantage over the other chairs was observed. This suggests that the high ergonomic risk is primarily influenced by the working posture required for viewing the patient’s mouth rather than by the body position induced by the chair’s design [32,36].

In order to facilitate a comparison of the joint angles with the specifications of an ideal sitting posture, reference should be made to the ISO standard 11226 [37] on ergonomics and the evaluation of postures at work. The ideal seated posture is upright and symmetrical [37]. The European Society of Dental Ergonomics (ESDE) further recommends minimising upper arm movement and leaning against the upper body to maintain a stable, active working posture [38,39]. However, an ergonomic risk assessment of the current working posture indicates moderate to severe alterations. Regardless of the type of work chair, the risk of developing workplace-related musculoskeletal disorders is always present. To date, there have been a limited number of studies that have examined the impact of chair design on the posture of dental professionals, as evaluated using the RULA ergonomic risk assessment [8,9,10,11,12,13,14]. However, when such studies have been conducted, they have predominantly utilised the conventional paper–pencil method [13]. For instance, Dable et al. [13] analysed the ergonomic saddle chair from Salli Systems (Easydoing OY/Salli Systems, Rautalampi, Finland) and two conventional chairs with a horizontal seat, and they were able to demonstrate a significantly lower RULA total score of the right and left side of the body of approximately three score points between the ergonomic saddle chair and the other two chairs. A similar conclusion was reached by Gandavadi et al. [14] with a lower score of approximately 2.5 score points. Both saddle chairs used here are designed according to the same principle as the saddle chair from the company XO in the present study with a hip–knee angle of 135° [40].

The posture on the saddle chair, which was assessed as lower risk, could not be confirmed. Although the XO saddle chair tended to have the lowest overall score of five, there was no statistically significant difference compared to other chair types. Possible reasons for deviating results from the studies by Dable et al. [13] and Gandavadi et al. [14] could be methodological differences and different participant groups. While dental students without ergonomic training were examined in these studies, the licenced dentists in this study can be assumed to have the corresponding ergonomic expertise. In addition, earlier studies were based on snapshots (using the conventional RULA paper–pencil method), whereas in this study, continuous, kinematic data collection was used, which made it easier to record differences in load over time [41].

The application of alternative measurement methods, such as video stereography [12] or EMG measurements [11], failed to identify any disparities in the posture of the upper body in both habitual and simulated working postures when comparing the various ergonomic layouts of the task chairs. Additionally, no significant findings were observed in the muscle groups associated with the spine [11]. Despite the use of different measurement methods, these findings confirm the existing results. In contrast, other studies have indicated an influence of ergonomically shaped work chairs on the muscle activity of various postural muscles compared to conventional chairs [9,42,43,44]. The activity of the trapezius muscle [42,43,44], the erector spinae muscle [42], and the deltoid muscle [43] was reduced by ergonomic task chairs, but this influence cannot be transferred to the joint angles. The differences in joint angles on the various work chairs are either very marginal and rarely recorded, or they have little or no influence on the ergonomic assessment.

These methodological challenges are also confirmed by other studies of the SOPEZ project [6] in which comparably high RULA scores were found in dentists of different specialities [36], including endodontists with magnifying loupes and XO saddle chairs [23]. Continuous recording allows conclusions to be drawn about the angular range and the relative frequency of joint angles and joint positions over a defined period of time and not just a single image as with the original RULA method.

The biomechanical measurement was conducted within a laboratory setting, thereby precluding the option of conducting the measurement on the practice’s own premises. This was due to logistical and practical constraints, which resulted in the emphasis being placed on ensuring internal validity. This approach entailed the execution of all the tasks on a dummy head with plastic teeth, thereby minimising the potential influence of external patient-specific factors on posture. In addition, real treatment cases would have been difficult to compare, as anatomical conditions (oral cavity size, saliva flow, and treatment complexity) and individual patient needs (breaks due to mouth closure and limitations in positioning) vary significantly and influence the working posture of dental practitioners. This standardisation created ideal experimental conditions, minimising the potential impact of external patient-specific factors on posture.

Furthermore, the high internal validity of the study enabled a comparison of the work chairs [45]. The reliability and accuracy of inertial measurement systems have been confirmed by various studies [46,47,48,49], and the validity and reliability of the inertial measurement system used in this study have also been confirmed [50,51].

A further limitation of the study is the standardisation of the sitting position based on a knee joint angle of 120°, which ensures high internal validity. However, the dentists were not permitted to adopt a habitual sitting height, which reduces the external validity, as the results may not be transferable to real work situations. This change in sitting height could have influenced the posture of the participants and negatively affected the chair comparison. Furthermore, individual differences in dentists’ physical conditions may have introduced additional variability, potentially skewing the results. In future studies, it would be beneficial to incorporate measures of muscle activity and to consider the deterioration of posture throughout the workday, as fatigue has been shown to influence posture, and consequently the position of the lumbar spine in the sagittal plane.

In conclusion, no ergonomic differences were observed among the dental chairs examined in this study, as indicated by the identical RULA scores during dental activities on the dummy head. Consequently, it can be inferred that each dentist should select their chair based on subjective comfort preferences. However, our study does not assume that only the design of the chair alone has an influence on the ergonomic risk,

In addition to the chair design, the ergonomic risk, determined using RULA, of different dental work concepts during various dental activities from different areas of dentistry, such as conservative dentistry, orthodontics or endodontology, is almost equally high [21,22,23,36]. Instead, other preventive measures such as magnifying glasses, ergonomic training or strength training [2,8,20] should be considered as practical recommendations for reducing MSDs, As part of the SOPEZ project [6], it was found that a 10-week resistance training programme for dentists and dental assistants reduces the intensity of pain (particularly in the trunk region) and improves the maximum voluntary isometric contraction of the trunk muscles, but does not significantly affect dental work activities (RULA score remains high) [20]. It is, therefore, suitable as a behavioural preventive measure against MSDs in dentists and dental assistants.

Another factor that should also have been taken into account is a lack of adaptation period to the chairs and the dental work concept. However, as all the participants work with the same dental work concept in the practice, familiarity can be assumed in advance. With regard to the chairs, one of the five chairs used by all the participants was also the one they use in everyday dental practice. In future studies, each subject could be given sufficient time to familiarise themselves with all the chairs. This would help to isolate the immediate effects of the chair design from long-term comfort and ergonomic benefits.

The fatigue of the muscles over the course of a working day and whether a ‘postural deterioration’ can be recognised as a result is another factor to be discussed. Due to the duration of our measurement protocol of 30–40 min, it was not possible to record fatigue and the associated possible postural deterioration.

In addition, electromyography to record muscle activity could also have provided revealing information here. This would also have been interesting with regard to aspects of muscle strain or the reaction of the muscles due to prolonged sitting or poor posture. The absence of these data limits the ability to assess the actual physical demands placed on dental practitioners while using the chairs. Therefore, future studies should combine kinematic data with EMG, which would allow for consideration of fatigue effects and for a better understanding of the muscle groups most affected by different chair designs and could lead to more specific ergonomic recommendations.

## 5. Conclusions

This study is the first to compare ergonomic postures during dental treatment on different dental chairs using quantitative data collection and ergonomic analysis. Overall, the results of the RULA method show that there are no significant ergonomic differences in posture between the dental chairs tested. Nevertheless, the median RULA scores for the right and left sides of the dentists’ bodies were between 5 and 6, indicating a high risk of developing musculoskeletal disorders. However, this analysis does not indicate whether the isolated differences in joint angles have an impact on ergonomics or represent an increased risk. It should be noted that most of the significant differences in the frequency of joint angles are within a RULA threshold.

Overall, the results confirm that the dentist’s working posture has an influence on the risk of musculoskeletal disorders and that the ergonomic design of the dental chair plays a minor role.

## Figures and Tables

**Table 1:** Characteristics of the six chairs used: special attributes, sitting area, and height of sitting position.

Chair	Company, Chair Name	Special Attributes/ Backrest	Sitting Area	Height of Sitting Position (Knee Ankle)
Chair 1	Dentsply Sirona (Germany), CARL	the backrest can be swivelled through 360°	disc-shaped and horizontal	height adjustable
Chair 2	Newberg Oregon, USA, A-dec	adjustable backrest angle and height	adjustable seat inclination	height adjustable
Chair 3	Veenendaal, Netherlands, Ghopec BV		rear section is horizontal and the front section is adjustable in inclination	seat height adjustable via a lever the angle between the hip and knee should be at least 110° for tall people and a maximum of 135° for short people
Chair 4	Hørsholm, Denmark, XO- Saddle chair	no backrest	saddle-shaped, two parts mutually adjustable, and adjustable inclination	height adjustable manufacturer information: 135° knee angle
Chair 5	Haar, Germany, Aeris, Swopper	no backrest	hemispherical, resilient, and flexible	height adjustable, weight adjustable, spring tension adjustable, and lateral deflection adjustable

**Table 2:** Standardised task protocol for each quadrant.

Task	1. Quadrant Filling on Tooth 17	2. Quadrant Dental Calculus Removal	3. Quadrant Crown Preparation on Tooth 35	4. Quadrant Root Canal Treatment on Tooth 46
1	Preparation of a cavity and removal of the contact point with a diamond-coated grinding wheel	Removal of supra- and subgingival calculus with scalers and curettes	Occlusal reduction with a diamond-coated bur	Trepanation of the tooth with a diamond-coated grinding tool
2	Position the Tofflemire matrix and seal the gap with a wedge		Circumferential grinding of the tooth with a torpedo-shaped grinding instrument	Locate and visualise the three root canals with hand instruments
3	Filling the cavity with Ketac^®^ and modelling with hand instruments			Preparation of canals with hand instruments ISO15-40 and irrigation of canals with irrigation fluid

**Table 3:** Median RULA scores and *p*-value of the conducted Friedman test after Bonferroni correction for each body region. The maximum achievable RULA score is indicated in parentheses behind the respective body region. The interquartile distance is indicated in parentheses behind the median RULA scores.

Body Region	Carl	A-dec 500	Ghopec	XO	Swopper	*p*-Value
Final left (7)	5 (1)	5.5 (1)	6 (0.75)	5 (1)	5 (1)	0.153
Final right (7)	5.5 (1.5)	6 (1)	5.5 (1)	5 (0.75)	5.5 (1)	0.091
Neck (6)	3.5 (1)	3.5 (1)	3.5 (0.75)	3.5 (1)	3.5 (0.5)	0.617
Upper Body (6)	3 (1)	3 (0.5)	3 (1)	2.5 (1)	2.5 (1)	0.380
Upper Arm left (6)	1 (0.5)	1 (0.5)	1.5 (0.75)	1 (0.5)	1(0.75)	0.273
Lower Arm left (3)	3 (0.5)	3 (0.75)	3 (0.5)	3 (1)	3 (0.5)	0.591
Wrist left (6)	4.5 (0.75)	4.5 (0.5)	4.5 (1)	4.5 (1)	4.5 (1)	0.054
Upper Arm right (6)	2 (0)	2 (0.25)	2 (0.25)	2 (0.5)	2 (0)	0.478
Lower Arm right (3)	2 (0.5)	2 (0.75)	2 (0.5)	2 (0.75)	2 (1)	0.941
Wrist right (6)	4.5 (1)	4.5 (1)	4.5 (1)	5 (1)	4.5 (1)	0.316

## Data Availability

The raw data supporting the conclusions of this article will be made available by the authors on request.
